# Diagnosis of Second Mesiobuccal Canal in Maxillary First Molars among Patients Visiting a Tertiary Care Hospital

**Published:** 2015-06

**Authors:** Muhammad Hasan, Farhan Raza Khan

**Affiliations:** 1 Department of Surgery, The Aga Khan University Hospital, Stadium Road, Karachi, Pakistan;; 2 Dental Section, Aga University Hospital, Karachi, Pakistan

**Keywords:** Mesiobuccal canal, maxillary first molar, magnification

The mesiobuccal root of the maxillary first molar has generated more research and clinical investigation than any other root. An inability to detect and treat a second mesiobuccal canal (MB2) is a reason for endodontic failure in maxillary first molars (1). Modifications in endodontic access and detection techniques, along with advancements in illumination and magnification technology, have aided in the location and treatment of the second mesiobuccal canal of maxillary first molars (2). Studies have shown an incidence of MB2 in maxillary first molars to be 63% (3).

The objective of our study was to determine the frequency of the second mesiobuccal canal in the permanent maxillary first molars with magnification loupes (× 3.5). In this cross sectional study, a total of 53 teeth were assessed clinically using magnification loupes for MB2 canal in mesiobuccal root of first maxillary molars. Detection of MB2 canal was done through a clinical access cavity preparation with magnification (× 3.5) with controlled pulp chamber floor troughing. We obtained institution ethical board’s clearance for this study (Ref: 1567-Sur-ERC-2010).

We were able to detect MB2 in 27 out of 53 (50.9%) of maxillary first molars. It was found that males tend to have a higher proportion of MB2 canals up to 31% as compared to females in whom the MB2 could be identified only 19% of the time.

Weine FS *et al*. were the first to report that clinician’s inability to locate and fill the second mesiobuccal canal can result in an endodontic failure in maxillary molars (4). This may be attributed to the anatomical diversity in MB2 canal system which invariably originates within the sub-pulpal groove connecting the two main canals, making its detection challenging (5).

**Figure 1:**
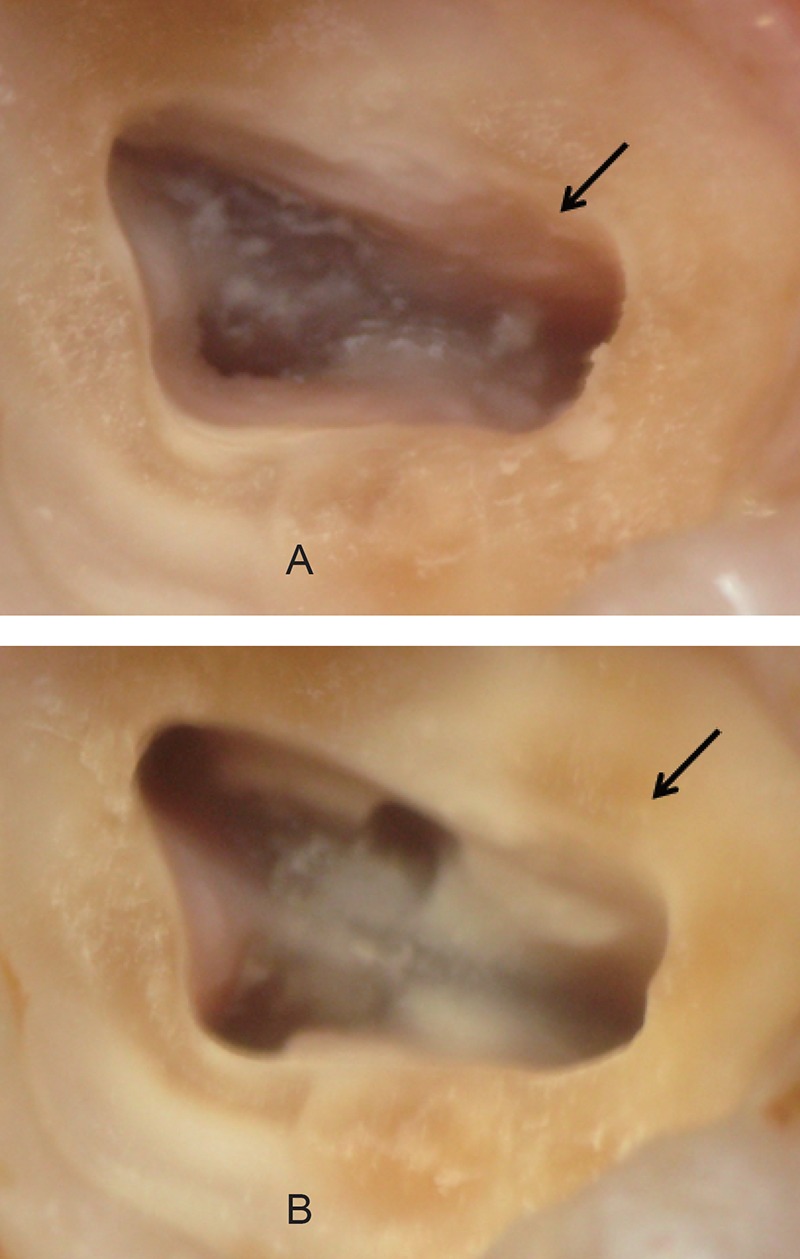
Clinical case demonstrating initial access opening and identification of the developmental groove between the palatal and primary mesiobuccal canals [black arrow] [A], Controlled chamber floor troughing lead to revelation of the second mesiobuccal canal [black arrow] [B].

Within the limitations of our study, we conclude that the use of the magnification loupes (× 3.5) enhanced the detection (50.9%) of the MB2 canals in the maxillary first molars. In addition, the prudent clinician is suggested to employ the canal-search strategies such as chamber floor troughing, assessment of radiographic width of the mesiobuccal root, CEJ perimeter, tooth angulation, cusp tip-pulp floor distance (CPFD), and the laws of orifice location to successfully locate the second mesiobuccal canal system to improve the outcome of endodontic treatment (Figure 1).
